# Self-Assembly of Bottlebrush Block Copolymers in Selective Solvent: Micellar Structures

**DOI:** 10.3390/polym13091351

**Published:** 2021-04-21

**Authors:** Inna O. Lebedeva, Ekaterina B. Zhulina, Oleg V. Borisov

**Affiliations:** 1 Institut des Sciences Analytiques et de Physico-Chimie pour l’Environnement et les Matériaux, UMR 5254 CNRS UPPA, 64000 Pau, France; innale92@gmail.com; 2 Institute of Macromolecular Compounds of the Russian Academy of Sciences, 190121 St. Petersburg, Russia; kzhulina@hotmail.com

**Keywords:** block copolymers, bottlebrushes, self-assembly

## Abstract

Block copolymers comprising chemically different bottlebrush blocks can self-assemble in selective solvents giving rise to micellar-like solution nanostructures. The self-consistent field theoretical approach is used for predicting relation between architectural parameters of both bottlebrush blocks (polymerization degrees of the main and side chains, density of grafting of the side chains to the backbone) and structural properties of micelles as well as critical micelle concentration (CMC). As predicted by the theory, replacement of linear blocks by bottlebrush ones with the same degrees of polymerization results in a decrease in the micellar core size (in aggregation number) and extension of the corona, whereas the CMC increases. These theoretical findings are in good agreement with results of computer simulations.

## 1. Introduction

Diblock copolymers comprising chemically different blocks *A* and *B* are capable of self-assembly in selective solvent, which is good for blocks *A*, but poor for block *B*, thus giving rise to nanoscale micellar-like aggregates. In such aggregates, insoluble *B* blocks associate into solvent-free core domain decorated by solubilizing corona formed by solvophilic *A* blocks. Amphiphilic diblock copolymers with hydrophobic *B* block and hydrophilic (neutral or ionically charged) *A* blocks represent generic type of polymeric surfactants resembling in many aspects their low molecular weight counterparts [1].

Rational understanding of self-assembly of block copolymers in selective solvent has been achieved on the basis of existing theories [2,3,4,5,6,7] amply supported by experiments (see, e.g., reviews [8,9]). A generic feature of block copolymer assembly is coupling of inter-molecular association to conformational changes in both blocks which makes self-assembly of block copolymers more complex than that of conventional surfactants. Structure and properties of self-assembled aggregates can be efficiently controlled by DPs of blocks, as well as by tuning their solubility (so called stimuli-responsiveness) [6].

Moreover, as has been recently understood, by changing topology (mode and degree of branching) of the blocks, one can achieve new properties of the self-assembled nanostructures without affecting chemical nature of the constituent blocks. Linear-dendritic or double dendritic block copolymers (termed also as amphiphilic or Janus dendrimers) represent typical examples [10,11,12,13,14,15]. Such copolymers are most promising for biomedical applications due to large number of exposed to environment and potentially functionizable terminal groups of dendritically-branched solvophilic blocks [16].

Recently, self-assembled structures of block copolymers with bottlebrush-like blocks have attracted considerable interest. Amphiphilic diblock copolymers with bottlebrush soluble blocks can replace in many aspects copolymers with dendritic blocks because they comprise large number of functionizable end segments in multiple lateral branches. Moreover, triblock copolymers with middle bottlebrush blocks can associate in solution or in the melt state giving rise to materials (microphase segregated, mesogels) with unique mechanical properties resembling biological tissues and efficiently controllable through the generic set of architectural parameters, i.e., degrees of polymerization of the main and side chains and grafting density in the bottlebrush blocks.

Although the first computer simulations [17,18] proved the possibility of affecting the micellar structure by varying architecture of the bottlebrush blocks, theoretical understanding of systematic relations between the topological parameters set and experimentally relevant properties (aggregation number, overall dimensions) of micelles formed by bottlebrush block copolymers is still missing. Here, we aim to fill this important knowledge gap. The aim of the present paper is to develop a theory of self-assembly of diblock copolymers comprising one soluble and one insoluble bottlebrush blocks in selective solvent (linear-bottlebrush block copolymers represent a particular case).

## 2. Model and Method

**Figure 1:**
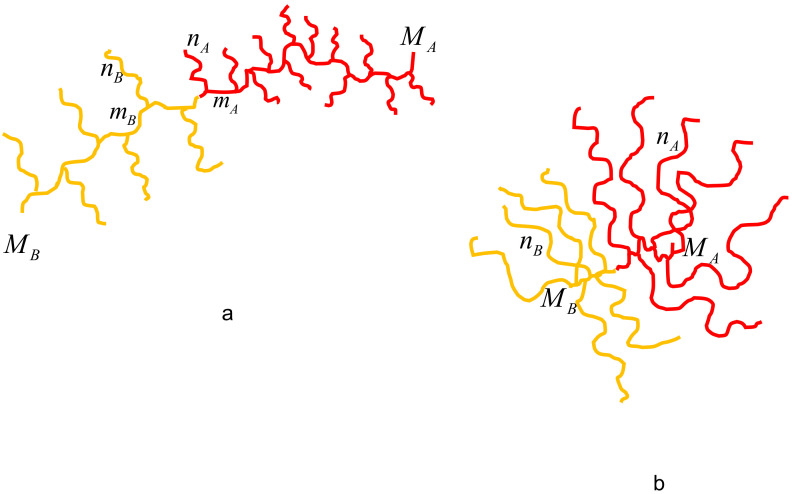
Schematics of bottlebrush (**a**) and miktoarm star (**b**) diblock copolymer for a particular case of branching activity $q_{A}=q_{B}=1$.

We consider diblock copolymers comprising two chemically different comb-shaped blocks in selective solvent, Figure 1. Block *A* has total degree of polymerization (DP) $N_{A}$, the DP of the main chain $M_{A}$, DPs of side chains $n_{A}$, and number of monomers in a spacer separating two neighboring grafting points $m_{A}$. The number of side chains emanating from each branching point is $q_{A}$. The ratio $q_{A}/m_{A}$ (number of side chains per monomer unit of the main chain) quantifies grafting density. Total number of side chains in the block A is $M_{A}q_{A}/m_{A}$. The set of architectural parameters for the block *A* will be abbreviated as $\left\{M_{A};n_{A};m_{A};q_{A}\right\}$. Block *B* with total DP $N_{B}$ is characterized by the corresponding set of parameters $\left\{M_{B};n_{B};m_{B};q_{B}\right\}$. Below we focus on comb-shaped copolymers with densely grafted side chains, $q_{A,B}n_{A,B}/m_{A,B}\gg 1$, and, following standard nomenclature, term them as molecular bottlebrushes. Evidently, $n_{A}=0$ or $n_{B}=0$ correspond to linear respective blocks. The DPs of the blocks are expressed through the sets of architectural parameters as

(1) $$N_{i}=M_{i}\left(1+\frac{q_{i}\cdot n_{i}}{m_{i}}\right)\;i=A,B$$

The backbone and the side chains in each of the blocks are assumed to be chemically identical, the monomer unit size *a* (the same for both blocks), is on the order of the Kuhn segment length. The solvent is assumed to be poor for monomer units of blocks *B* and moderately good for monomer units of blocks *A*.

Poor solubility of blocks *B* drives association of block copolymers in selective solvent that leads to formation of micellar-like aggregates where blocks *B* constitute solvent-free core domain whereas soluble blocks *A* protrude in solution to form corona. Here, we focus on spherical micelles although depending on the DPs of blocks *A* and *B*, and their degrees of branching other morphologies (wormlike micelles, polymersomes) may correspond to thermodynamic equilibrium structures which will be considered elsewhere.

Poor solubility of blocks *B* assures narrow core–corona interface so that the corona and the core of micelle can be envisioned as convex swollen and concave dry brushes of *A* and *B* blocks tethered to the core–corona (A/B) interface, respectively.

We implement the strong stretching self-consistent field (SS-SCF) approximation for calculating the free energies of both corona (*A*) and core (*B*) domains. In the SS-SCF formalism, the self-consistent molecular potential acting on monomer units of *A* or *B* blocks is parabolic and presented [19,20,21] as

(2) $$\frac{U_{i}\left(z\right)}{k_{B}T}=\frac{3\kappa_{i}^{2}}{2a^{2}}\cdot \left\{\begin{array}{cc} D^{2}-z^{2}, & i=A\\ R^{2}-z^{2}, & i=B \end{array}\right.$$

Here, *D* and *R* are the corona thickness and radius of the core, respectively (R+D is overall radius of micelle), z∈[0,D] or z∈[0,R] are the distances from the core–corona interface, $k_{B}T$ is the thermal energy, and i=A,B.

The topological coefficients $\kappa_{i}$ depend on the set of topological parameters $\left\{M_{i},n_{i},m_{i},q_{i}\right\}$ of each respective block. Below, instead of the topological coefficients $\kappa_{i}$, we use the topological ratios

(3) $$\eta_{i}=\frac{2N_{i}\kappa_{i}}{\pi }$$

which are equal to unity for linear blocks (“bare backbones”), $N_{i}=M_{i}$ at $n_{i}=0$ but are larger than unity for any branched, e.g., bottlebrush blocks. The topological ratio quantifies the relative increase in the elastic free energy per chain in the brush formed by branched macromolecules as compared to that in the brush formed by linear chains with the same degree of polymerization. The topological ratio depends on the macromolecular architectural parameters but is independent of the chain grafting density, and the character of intermolecular interactions in the brush.

The topological ratio for brushes of comblike macromolecules was calculated in ref. [22] and can be expressed analytically in the two asymptotic limits

(4) $$\eta_{i}\approx \left\{\begin{array}{cc} {\left(1+q_{i}n_{i}/m_{i}\right)}^{1/2}, & M_{i}\gg {\left(n_{i}m_{i}/q_{i}\right)}^{1/2}\\ q_{i}M_{i}/m_{i}, & M_{i}\ll {\left(n_{i}m_{i}/q_{i}\right)}^{1/2} \end{array}\right.$$

The first line in Equation (4) corresponds to the comblike block with long backbone and multiple relatively short side chains attached to it (bottlebrush limit, as depicted in Figure 1a), whereas the second line corresponds to the block with few long arms attached to the relatively short backbone (miktostar limit, depicted in Figure 1b). Although the topological coefficient (and the topological ratio) can be calculated for comb-shaped blocks with arbitrary set $\left\{M_{i},n_{i},m_{i},q_{i}\right\}$ of the parameters, the self-consistent potential exhibits the parabolic dependence on *z*, Equation (2), provided (i) Gaussian conformational elasticity of all linear segments of the branched blocks on all length scales and (ii) absence of “dead zone” proximal to A/B interface and depleted from the free ends of backbones of blocks. In convex spherical geometry the latter criterion can be violated but, nonetheless, Equation (2) with topological ratio given by Equation (4) can serve as a reasonable approximation.

In this paper, we focus on copolymers with comb-shaped blocks that exhibit linear (Gaussian) elasticity on all length scales. Although, due to repulsive interactions between densely grafted side chains, the backbones of comb-shaped polymers in the melt or/and in solution are elongated with respect to unperturbed Gaussian end-to-end dimensions [23], the application of the SS-SCF approach to micellar corona and core ignores pre-stretching of backbones in the individual A and B blocks. A more elaborated model taking into account renormalization of the blocks’ elasticity will be considered elsewhere.

Below we analyze how parameters of micelles (aggregation number, radius of the core, corona thickness, and the overall radius of the micelle) depend on the length of side chains $n_{i}$ for $N_{i},m_{i}=const$ or $M_{i},m_{i}=const$. For that, Equation (4) can be presented as

(5) $$\eta_{i}\left(n_{i}\right)\approx \left\{\begin{array}{cc} {\left(1+q_{i}n_{i}/m_{i}\right)}^{1/2}, & n_{i}\ll N_{i}^{2/3}{\left(m_{i}/q_{i}\right)}^{1/3}\\ N_{i}/\left(n_{i}+m_{i}/q_{i}\right), & n_{i}\gg N_{i}^{2/3}{\left(m_{i}/q_{i}\right)}^{1/3} \end{array}\right.$$

or

(6) $$\eta_{i}\left(n_{i}\right)\approx \left\{\begin{array}{cc} {\left(1+q_{i}n_{i}/m_{i}\right)}^{1/2}, & n_{i}\ll M_{i}^{2}q_{i}/m_{i}\\ q_{i}M_{i}/m_{i}, & n_{i}\gg M_{i}^{2}q_{i}/m_{i} \end{array}\right.$$

Obviously, $n_{i}$ is limited from above as $n_{i}\leq N_{i}-m_{i}$.

Alternatively, we can analyze parameters of micelles as a function of grafting density $m_{i}/q_{i}$ for $N_{i},n_{i}=const$ or $M_{i},n_{i}=const$, so that

(7) $$\eta_{i}\left(m_{i}/q_{i}\right)\approx \left\{\begin{array}{cc} {\left(1+q_{i}n_{i}/m_{i}\right)}^{1/2}, & m_{i}/q_{i}\gg N_{i}^{-2}n_{i}^{3}\\ N_{i}/\left(n_{i}+m_{i}/q_{i}\right), & m_{i}/q_{i,}\ll N_{i}^{-2}n_{i}^{3} \end{array}\right.$$

or

(8) $$\eta_{i}\left(m_{i}/q_{i}\right)\approx \left\{\begin{array}{cc} {\left(1+q_{i}n_{i}/m_{i}\right)}^{1/2}, & m_{i}/q_{i}\ll M_{i}^{2}n_{i}^{-1}\\ M_{i}q_{i}/m_{i}, & m_{i}/q_{i}\gg M_{i}^{2}n_{i}^{-1} \end{array}\right.$$

The first and the second lines in Equations (5)–(8) refer to bottlebrush or miktostar shape of a block, respectively.

## 3. Free Energy of a Micelle

The properties of equilibrium micelles are obtained through minimization of the free energy per block copolymer molecule,

(9) $$F=F_{corona}+F_{core}+F_{interface}$$

which comprises contribution of the corona, $F_{corona}$, and the core, $F_{core}$, domains, as well as the free energy of the core–corona interface, $F_{interface}$.

Within SS-SCF approximation, the self-consistent molecular potential acting on the monomers of corona chains is given by Equation (2), and related to the interaction free energy density $f_{int,A}\left\{\phi_{A}\left(z\right)\right\}$ as

$$U\left(z\right)=\frac{\delta f_{int,A}\left\{\phi_{A}\left(z\right)\right\}}{\delta \phi_{A}\left(z\right)}$$

where $\phi_{A}\left(z\right)$ is concentration (volume fraction) profile of monomer units of block *A* in the corona.

Under good solvent conditions,

(10) $$f_{int,A}\left\{\phi_{A}\left(z\right)\right\}=k_{B}Ta^{-3}v\phi^{2}\left(z\right)$$

where *v* is dimensionless (normalized by $a^{3}$) second virial coefficient, and, therefore

(11) $$\phi_{A}\left(z\right)=\frac{3\kappa_{A}^{2}}{4va^{2}}\left(D^{2}-z^{2}\right)$$

also exhibits a parabolic shape.

Detailed calculations of the free energies of corona and core domains in a micelle formed by copolymers with arbitrary branched architecture of blocks are presented elsewhere [15] and here we outline only the key steps.

The cumulative free energy of the corona and the core domains can be presented as

$$F_{corona}+F_{core}=$$

(12) $$\int_{0}^{D}f_{int,A}\left\{\phi_{A}\left(z\right)\right\}s_{A}\left(z\right)dz+\int_{0}^{D}f_{elastic,A}\left(z\right)s_{A}\left(z\right)dz+\int_{0}^{R}f_{elastic,B}\left(z\right)s_{B}\left(z\right)dz$$

where $f_{int,A}\left\{\phi_{A}\left(z\right)\right\}$, $f_{elastic,A}\left(z\right)$ and $f_{elastic,B}\left(z\right)$ are the free energy of excluded volume interactions, the elastic (conformational) free energy per unit volume in the corona, and of the core, respectively. Here *s* is the core surface area per block copolymer molecule and $s_{A,B}\left(z\right)$ is defined as

(13) $$s_{i}\left(z\right)=s\cdot {\left(\frac{R\pm z}{R}\right)}^{2}$$

where signs “+” and “−” refer to the corona (i=A) and core (i=B) domains, respectively

The condition of uniform packing of blocks *B* in the core imposes the relation between the core–corona A/B interface area *s* per block copolymer and the radius *R* of the core domain

(14) $$s=\frac{3N_{B}a^{3}}{R\phi_{B}},$$

The number *p* of block copolymer chains in a spherical micelles is related to the radius *R* of the core or to the core surface area *s* per copolymer chain as

(15) $$p=\frac{4\pi R^{3}\phi_{B}}{3N_{B}}=\frac{36\pi N_{B}^{2}a^{6}}{s^{3}\phi_{B}^{2}}$$

where $\phi_{B}≃1$ is the volume fraction of monomer units *B* in the core.

Conservation of the number of monomer units of the *A* blocks, $\int_{0}^{D}A\left(z\right)s_{A}\left(z\right)dz=N_{A}a^{3}$, provides (with account of Equation (11)) the relationship between s,R and *D*

(16) $$D=D_{0}\left(s\right)\cdot {\left(1+\frac{3}{4}\frac{D}{R}+\frac{1}{5}\frac{D^{2}}{R^{2}}\right)}^{-1/3}$$

where

(17) $$D_{0}\left(s\right)={\left(\frac{2N_{A}va^{2}}{s\kappa_{A}}\right)}^{1/3}a\equiv {\left(\frac{8}{\pi^{2}}\right)}^{1/3}N_{A}v^{1/3}{\left(\frac{a^{2}}{s}\right)}^{1/3}\eta_{A}^{-2/3}a$$

is the thickness of a planar brush (with zero curvature) with grafting area *s* per molecule.

As long as both blocks *A* and *B* exhibit Gaussian elasticity, the density of elastic free energy can be expressed as

(18) $$f_{elastic,i}\left(z\right)=\frac{T_{i}\left(z\right)}{2}$$

where $T_{i}\left(z\right)$ is the flux of elastic tension per unit area in the corresponding domains at distance *z* from A/B interface.

It can be presented as

(19) $$\frac{T_{i}\left(z\right)}{k_{B}T}=\frac{3\kappa_{i}^{2}}{2s_{i}\left(z\right)a^{3}}\int_{z}^{D,R}z^{\prime }\phi_{i}\left(z^{\prime }\right)s_{i}\left(z^{\prime }\right)dz^{\prime }$$

where the upper limits of the integral are equal to *D* or *R* for the corona and core domains, respectively, and $\phi_{A}\left(z\right)$ is given by Equation (11), while $\phi_{B}\left(z\right)\approx const≅1$.

After performing integration, one gets

(20) $$F_{corona}=F_{corona}^{\left(0\right)}\left(s\right){\left(\frac{D}{D_{0}\left(s\right)}\right)}^{5}\cdot \left(1+\frac{5}{6}\frac{D}{R}+\frac{5}{21}\frac{D^{2}}{R^{2}}\right)$$

where

(21) $$F_{corona}^{\left(0\right)}\left(s\right)=k_{B}T\frac{9}{20}\frac{\kappa_{A}^{4}s}{va^{2}}{\left(\frac{D_{0}\left(s\right)}{a}\right)}^{5}=k_{B}T\frac{9\pi^{2/3}}{10}N_{A}v^{2/3}{\left(\frac{a^{2}}{s}\right)}^{2/3}\eta_{A}^{2/3}$$

is the free energy (per molecule) in a planar brush with area *s* per *A* block and Equation (3) for $\eta_{A}$ was used.

The elastic free energy of block *B* in the core with radius *R* can be expressed using Equations (12) and (19) as

(22) $$F_{core}\left(s\right)=\int_{0}^{R}f_{elastic,B}\left(z\right)s_{B}\left(z\right)dz=k_{B}T\frac{27\pi^{2}}{80}\frac{N_{B}a^{4}}{s^{2}\phi_{B}^{2}}\eta_{B}^{2}$$

The excess free energy of the core–corona interface (per block copolymer chain) can be expressed as

(23) $$F_{interface}=k_{B}T\gamma s$$

where $\gamma k_{B}T$ is the interfacial free energy per unit area, which is controlled primarily by solubility parameter of the core-forming *B*-blocks but is virtually independent of concentration of monomer units of *A*-blocks in the corona domain.

Using Equations (14), (16), and (17), the area *s* of the core surface per block copolymer chain can be presented as

(24) $$\frac{s}{a^{2}}=\frac{\pi \eta }{N_{A}^{3/2}v^{1/2}}{\left(\frac{N_{B}}{\phi }\right)}^{3/2}\cdot {\left(\frac{3D}{2R}\right)}^{3/2}{\left(1+\frac{3}{4}\frac{D}{R}+\frac{1}{5}\frac{D^{2}}{R^{2}}\right)}^{1/2}$$

As follows from Equation (24), the surface area *s* per block copolymer chain is expressed as a function of dimensionless ratio

(25) x=D/R

The free energy of the core–corona interface is given by

(26) $$F_{interface}\left(x\right)=\frac{k_{B}T\gamma s}{a^{2}}=k_{B}T\gamma \frac{\pi \eta }{N_{A}^{3/2}v^{1/2}}{\left(\frac{N_{B}}{\phi }\right)}^{3/2}{\left(\frac{3x}{2}\right)}^{3/2}{\left(1+\frac{3}{4}x+\frac{1}{5}x^{2}\right)}^{1/2},$$

By substituting Equation (24) for *s* in Equations (12) and (21) for corona and core, contributions to the free energy can be also expressed as functions of *x*

(27) $$\frac{F_{corona}\left(x\right)}{k_{B}T}=\frac{3}{5}\frac{vN_{A}^{2}\phi }{N_{B}}\frac{\left(1+\frac{5}{6}x+\frac{5}{21}x^{2}\right)}{x{\left(1+\frac{3}{4}x+\frac{1}{5}x^{2}\right)}^{2}}$$

and

(28) $$\frac{F_{core}\left(x\right)}{k_{B}T}=\frac{N_{A}^{3}v\phi }{N_{B}^{2}}{\left(\frac{\eta_{B}}{\eta_{A}}\right)}^{2}\cdot \frac{1}{10}x^{-3}{\left(1+\frac{3}{4}x+\frac{1}{5}x^{2}\right)}^{-1}$$

By minimizing total free energy (per chain) as a function of *x*

(29) $$\frac{d[F_{corona}\left(x\right)+F_{interface}\left(x\right)+F_{core}\left(x\right)]}{dx}=0$$

which is equivalent to the minimization with respect to *s*, one finds the value of *s* (or *R*) corresponding to equilibrium micelle and, using Equations (9), (26)–(28) the free energy per block copolymer chain in the equilibrium micelle. This free energy is directly related to CMC, as

(30) $$k_{B}T\ln CMC=F\left(s\right)-F_{1}$$

where

(31) $$F_{1}\approx k_{B}T\gamma {\left(N_{B}/\phi_{B}\right)}^{2/3}$$

is (with the accuracy of numerical prefactor) the free energy of a unimer which is dominated by the excess free energy of the interface between single collapsed *B*-block and surrounding (poor) solvent. We remark that Equation (30) disregards translational entropy of micelles which is justified for sufficiently large aggregation numbers, p≫1.

## 4. Starlike and Crew-Cut Micelles: Asymptotic Results

**Figure 2:**
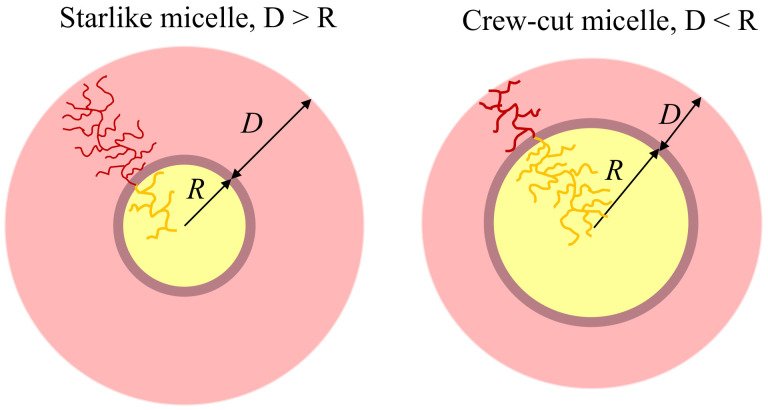
Schematics of starlike, D≫R, and crew-cut, D≪R, micelles formed by copolymers with bottlebrush blocks.

Following standard nomenclature, we distinguish starlike, D≫R, and crew-cut, D≪R, micelles (Figure 2). The overall dimensions of the micelles in these limiting cases are controlled either by the extension of the corona, *D*, or by the core radius, *R*, respectively. In both limits, structural and thermodynamic properties of micelles can be derived analytically in the form of power-law dependencies by keeping only the dominant terms in the free energy, that is, the free energy of the corona, $F_{corona}$ and the free energy of the core–corona interface, $F_{interface}$. Neglecting conformational entropy of the core-forming *B*-blocks does not allow us to capture the effect of their architecture on the properties of micelles, which may serve as a reasonable approximation as long as the *B*-blocks are linear or weakly branched. As demonstrated below, the calculations with proper account of the contribution of the conformational entropy of the *B*-blocks, prove that increasing branching of the *B*-blocks leads to systematic decrease in the dimensions and aggregation number of micelles. However, this decrease cannot be described in terms of power law dependencies on $\eta_{B}$.

The asymptotic power law dependencies of structural and thermodynamic properties of starlike and crew-cut micelles on the set $\left\{M_{A};n_{A};m_{A};q_{A}\right\}$ of architectural parameters of *A*-block and $N_{B}/\phi_{B}$ are presented below. They differ for the cases of bottlebrush, $M_{A}\gg {\left(n_{A}m_{A}/q_{A}\right)}^{1/2}$, and miktostar-like, $M_{A}\ll {\left(n_{A}m_{A}/q_{A}\right)}^{1/2}$, blocks *A*.

### 4.1. Starlike Micelles, D≫R

Aggregation number:

$$p≅\gamma^{15/11}{\left(\frac{N_{B}}{\phi_{B}}\right)}^{10/11}v^{-6/11}\cdot$$

(32) $$\left\{\begin{array}{cc} N_{A}^{-3/11}{\left(1+\frac{q_{A}n_{A}}{m_{A}}\right)}^{-9/11}=M_{A}^{-3/11}{\left(1+\frac{q_{A}n_{A}}{m_{A}}\right)}^{-12/11}, & M_{A}\gg {\left(n_{A}m_{A}/q_{A}\right)}^{1/2}\\ N_{A}^{-21/11}n_{A}^{18/11}{\left(1+\frac{m_{A}}{q_{A}n_{A}}\right)}^{18/11}={\left(\frac{M_{A}q_{A}}{m_{A}}\right)}^{-21/11}n_{A}^{-3/11}{\left(1+\frac{m_{A}}{q_{A}n_{A}}\right)}^{-3/11}, & M_{A}\ll {\left(n_{A}m_{A}/q_{A}\right)}^{1/2} \end{array}\right.$$

and core radius $R/a≅{\left(\frac{N_{B}}{\phi_{B}}\right)}^{1/3}p^{1/3}$.

Corona thickness:

$$D/a≅\gamma^{3/11}{\left(\frac{N_{B}}{\phi_{B}}\right)}^{2/11}v^{1/11}$$

(33) $$\left\{\begin{array}{cc} N_{A}^{6/11}{\left(1+\frac{q_{A}n_{A}}{m_{A}}\right)}^{-4/11}=M_{A}^{6/11}{\left(1+\frac{q_{A}n_{A}}{m_{A}}\right)}^{2/11}, & M_{A}\gg {\left(n_{A}m_{A}/q_{A}\right)}^{1/2}\\ N_{A}^{-2/11}n_{A}^{8/11}{\left(1+\frac{m_{A}}{q_{A}n_{A}}\right)}^{8/11}={\left(\frac{q_{A}M_{A}}{m_{A}}\right)}^{-2/11}n_{A}^{6/11}{\left(1+\frac{m_{A}}{q_{A}n_{A}}\right)}^{6/11}, & M_{A}\ll {\left(n_{A}m_{A}/q_{A}\right)}^{1/2} \end{array}\right.$$

The CMC:

$$\ln CMC≅-\gamma {\left(\frac{N_{B}}{\phi_{B}}\right)}^{2/3}+\gamma^{6/11}{\left(\frac{N_{B}}{\phi_{B}}\right)}^{4/11}v^{2/11}\cdot$$

(34) $$\left\{\begin{array}{cc} N_{A}^{1/11}{\left(1+\frac{q_{A}n_{A}}{m_{A}}\right)}^{3/11}=M_{A}^{1/11}{\left(1+\frac{q_{A}n_{A}}{m_{A}}\right)}^{4/11}, & M_{A}\gg {\left(n_{A}m_{A}/q_{A}\right)}^{1/2}\\ N_{A}^{7/11}n_{A}^{-6/11}{\left(1+\frac{m_{A}}{q_{A}n_{A}}\right)}^{-6/11}={\left(\frac{q_{A}M_{A}}{m_{A}}\right)}^{7/11}n_{A}^{1/11}{\left(1+\frac{m_{A}}{q_{A}n_{A}}\right)}^{1/11}, & M_{A}\ll {\left(n_{A}m_{A}/q_{A}\right)}^{1/2} \end{array}\right.$$

### 4.2. Crew-Cut Micelles, D≪R

Aggregation number:

$$p≅\gamma^{9/5}{\left(\frac{N_{B}}{\phi_{B}}\right)}^{2}v^{-6/5}\cdot$$

(35) $$\left\{\begin{array}{cc} N_{A}^{-9/5}{\left(1+\frac{q_{A}n_{A}}{m_{A}}\right)}^{-3/5}=M_{A}^{-9/5}{\left(1+\frac{q_{A}n_{A}}{m_{A}}\right)}^{-12/5}, & M_{A}\gg {\left(n_{A}m_{A}/q_{A}\right)}^{1/2}\\ N_{A}^{-3}n_{A}^{6/5}{\left(1+\frac{m_{A}}{q_{A}n_{A}}\right)}^{6/5}={\left(\frac{M_{A}q_{A}}{m_{A}}\right)}^{-3}n_{A}^{-9/5}{\left(1+\frac{m_{A}}{q_{A}n_{A}}\right)}^{-9/5}, & M_{A}\ll {\left(n_{A}m_{A}/q_{A}\right)}^{1/2} \end{array}\right.$$

and core radius $R/a≅{\left(\frac{N_{B}}{\phi_{B}}\right)}^{1/3}p^{1/3}$

Corona thickness:

$$D/a≅\gamma^{1/5}v^{1/5}\cdot$$

(36) $$\left\{\begin{array}{cc} N_{A}^{4/5}{\left(1+\frac{q_{A}n_{A}}{m_{A}}\right)}^{-2/5}=M_{A}^{4/5}{\left(1+\frac{q_{A}n_{A}}{m_{A}}\right)}^{2/5}, & M_{A}\gg {\left(n_{A}m_{A}/q_{A}\right)}^{1/2}\\ n_{A}^{4/5}{\left(1+\frac{m_{A}}{q_{A}n_{A}}\right)}^{4/5}, & M_{A}\ll {\left(n_{A}m_{A}/q_{A}\right)}^{1/2} \end{array}\right.$$

The CMC:

$$\ln CMC≅-\gamma {\left(\frac{N_{B}}{\phi_{B}}\right)}^{2/3}+\gamma^{2/5}v^{2/5}\cdot$$

(37) $$\left\{\begin{array}{cc} N_{A}^{3/5}{\left(1+\frac{q_{A}n_{A}}{m_{A}}\right)}^{1/5}=M_{A}^{3/5}{\left(1+\frac{q_{A}n_{A}}{m_{A}}\right)}^{4/5}, & M_{A}\gg {\left(n_{A}m_{A}/q_{A}\right)}^{1/2}\\ N_{A}n_{A}^{-2/5}{\left(1+\frac{m_{A}}{q_{A}n_{A}}\right)}^{-2/5}=\frac{q_{A}M_{A}}{m_{A}}n_{A}^{3/5}{\left(1+\frac{m_{A}}{q_{A}n_{A}}\right)}^{3/5}, & M_{A}\ll {\left(n_{A}m_{A}/q_{A}\right)}^{1/2} \end{array}\right.$$

## 5. Discussion

### 5.1. Diagram of States

**Figure 3:**
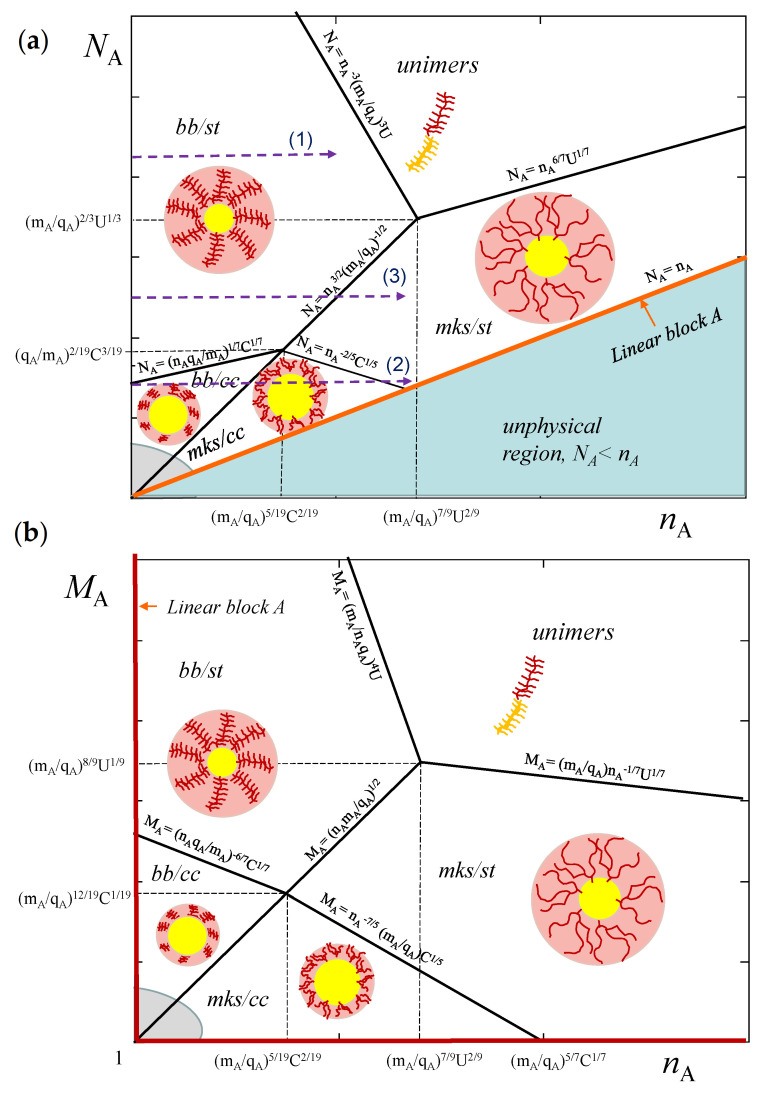
Schematic diagram of states of micelles in ($N_{A},n_{A}$) (**a**) and ($M_{A},n_{A}$) (**b**) coordinates. Regions of starlike (st) and crew-cut (cc) micelles with bottlebrush (bb) or miktostar (mks) A-blocks are outlined. Equations for boundaries between different regimes are presented near the lines. Parameters of the boundaries in the diagram are $C≅{\left(\gamma^{2}{\left(N_{B}/\phi_{B}\right)}^{5}v^{-3}\right)}^{1/7}$; $U=\gamma^{5}{\left(N_{B}/\phi_{B}\right)}^{10/3}v^{-2}$. Gray area corresponds to the region of spherical micelles instability (transition to wormlike micelles and polymersomes).

First of all, it is instructive to outline the ranges of stability of starlike (st) and crew-cut (cc) micelles with corona formed by copolymers with either bottlebrush (bb) or miktostar (mks) solvophilic blocks *A*. The corresponding diagrams of states are presented in ($N_{A},n_{A}$) and ($M_{A},n_{A}$) coordinates in Figure 3a,b, respectively. The diagrams contain regimes bb/st, mks/st, bb/cc, mks/cc of starlike or crew-cut micelles with bottlebrush and miktostar corona-forming A-blocks, respectively. The diagrams contain also the unimer regime where micellization is suppressed due to strong repulsive interactions between *A*-blocks. An increase in the overall DP of soluble blocks ultimately provokes stabilization of the unimer state of the block copolymers in the solution.

### 5.2. Structural and Thermodynamic Properties of Micelles

Below we discuss the main trends in the dependencies of the micellar properties (aggregation number, core, corona and overall dimensions, and CMC) on architectural parameters of the blocks. These trends are qualitatively captured by asymptotic Equations (32)–(37) whereas the explicit dependencies are accurately calculated at arbitrary ratio R/D according to the routine described in Section 3.

We start with analysis of the effect of varied side chains length, $n_{A}$, in soluble block *A* at either $N_{A},m_{A}/q_{A}=const$ or $M_{A},m_{A}/q_{A}=const$. An increase in $n_{A}$ (at $N_{A}=const$ or $M_{A}=const$) provokes transformation of the *A*-block from bottlebrush to miktostar-like and may also lead (particularly at $M_{A}=const$) to change of the shape of the micelles from crew-cut to starlike ones.

**Figure 4:**
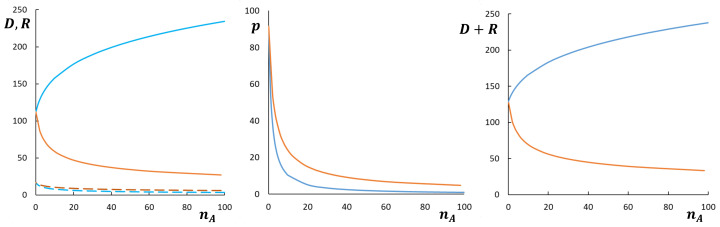
Aggregation number *p*, core radius *R* (dashed lines), and corona thickness *D* (solid lines), and overall radius, R+D, of starlike spherical micelles formed by block copolymers with $N_{B}=200$ and $N_{A}=1000$ or $M_{A}=1000$ as a function of $n_{A}$ (cross-section (1) in Figure 3a). Red and blue curves correspond to $N_{A}=1000=const$ (varied $n_{A},M_{A}$) or to $M_{A}=1000=const$ (varied $n_{A},N_{A}$), respectively. Other parameters are $q_{A}=1,m_{A}=2,\gamma =1,v=1,\phi_{B}=1$.

As illustrated by Figure 4, corresponding to cross-section (1) of the diagram in Figure 3a, at $N_{A},m_{A}/q_{A}=const$ the aggregation number, core radius, and corona thickness in starlike micelles with bottlebrush *A*-block decrease as a function of $n_{A}$. If not the total DP of the *A*-block, but rather DP of the main chain $M_{A}$ is kept constant, the aggregation number *p* and core radius *R* also decrease, whereas *D* and overall micellar size increase as a function of $n_{A}$. In both cases ($N_{A}=const$ or $M_{A}=const$) CMC is an increasing function of $n_{A}$.

**Figure 5:**
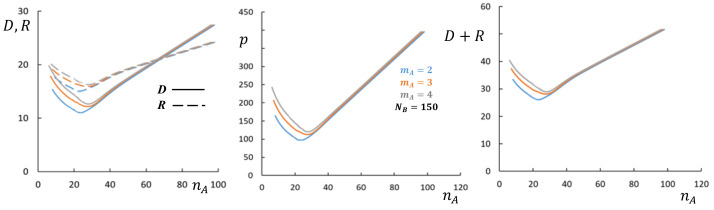
Aggregation number *p*, core radius *R* (dashed lines), and corona thickness *D* (solid lines), and overall radius, R+D, of micelles formed by block copolymers with $N_{B}=150$ and $N_{A}=100$ as a function of $n_{A}$ (cross-section (2) in Figure 3a). $m_{A}$ is varied, as indicated in the figure. Other parameters are $q_{A}=1,\gamma =1,v=1,\phi_{B}=1$.

Figure 5 demonstrates more complex, non-monotonous dependencies of the micellar parameters on $n_{A}$ at $N_{A}=const,m_{A}/q_{A}=const$ corresponding to cross-section (2) of the diagram in Figure 3a. Here aggregation number, core radius and corona thickness decrease as a function of $n_{A}$ as long as *A*-block is bottlebrush-like, but increase as a function of $n_{A}$ when *A*-block acquires miktostar shape. Hence, p,R,D, and R+D pass through a minimum as a function of $n_{A}$ when the shape of the coronal block *A* changes from bottlebrush to miktostar. In Figure 5, this crossover occurs in the regime of crew-cut micelles (R≥D), but similar trends are predicted for starlike micelles (cross-section (3) in Figure 3a). The CMC also depends non-monotonously on $n_{A}$, increasing in the range of bottlebrush *A*-blocks and decreasing in the range of miktostar *A*-blocks.

**Figure 6:**
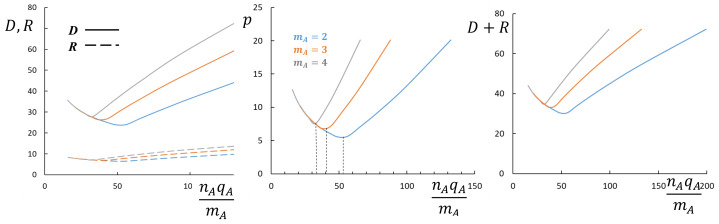
Aggregation number *p*, core radius *R* (dashed lines), and corona thickness *D* (solid lines), and overall radius, R+D, of micelles formed by block copolymers with $N_{B}=150$ and $N_{A}=100$ as a function of $n_{A}q_{A}/m_{A}$. $m_{A}$ is varied, as indicated in the figure. Other parameters are $q_{A}=1,\gamma =1,v=1,\phi_{B}=1$. The crossover between bottlebrush and miktostar shapes of the *A*-block is indicated by vertical dashed lines in the middle panel.

In Figure 6, the micellar structural properties p,R,D,R+D are plotted as a function of $q_{A}n_{A}/m_{A}$ at $N_{A}=const$ for a number of selected values of $q_{A}/m_{A}$. As one can see from the figure, all the dependencies follow universal curves as long as *A*-blocks keep bottlebrush shape but split into a series of curves each corresponding to specific value of $q_{A}/m_{A}$ at large $n_{A}$ when the *A*-block acquires miktostar shape.

**Figure 7:**
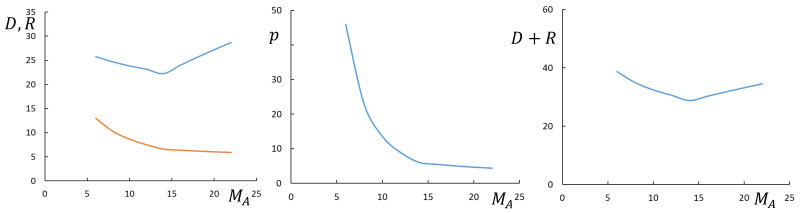
Aggregation number *p*, core radius, *R* (red), corona thickness, *D* (blue) and overall radius, R+D, of spherical micelles formed by block copolymers with $N_{B}=200$ as a function of $M_{A}$ at $n_{A}=100$. Other parameters are $q_{A}=1,\gamma =1,v=1,\phi_{B}=1$.

The effect of DP $M_{A}$ of the backbone in the corona block *A* (at constant $n_{A},m_{A}/q_{A}$) on the aggregation number and dimensions of micelles is as follows: As predicted by Equations (32), (33), (35), and (36) for both starlike and crew-cut micelles *p* and *R* do decrease as a function of $M_{A}$ while CMC increases. For starlike micelles, the corona thickness *D* and the overall micellar sise R+D increase as a function of $M_{A}$ in the case of bottlebrush A-block, but decrease in the case of miktoarm A-block. For crew-cut micelles, the corona thickness increases as a function of $M_{A}$ in the case of bottlebrush A-block, but is virtually independent of $M_{A}$ when *A*-block acquires miktostar shape. Hence, the corona thickness and the overall radius of the micelle may exhibit non-monotonic behavior as a function of $M_{A}$ passing through a minimum at the crossover between bottlebrush and miktostar regimes for the coronal block *A*, as illustrated by Figure 7 for crew-cut micelles.

**Figure 8:**
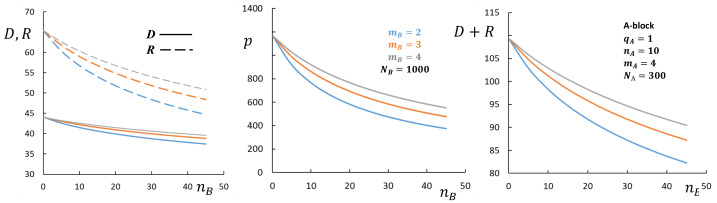
Aggregation number *p*, core radius, *R* (dashed lines), corona thickness, *D* (solid lines), and overall dimensions R+D as a function of the DP $n_{B}$ of side chains in the core forming block at $N_{B}=const=1000$. $m_{B}$ is varied, as indicated in the figure. Other parameters are $q_{A}=q_{B}=1$$N_{A}=300$, $m_{A}=4,n_{A}=10$γ=1,v=1,ϕ=1.

The effect of branching of the core-forming block *B* on the structural properties of spherical micelles is less pronounced, as illustrated in Figure 8. Although asymptotic Equations (32)–(37) neglecting conformational entropy of the core-forming blocks do not predict any dependence on the branching parameters of the core-forming B-blocks, detailed calculation with the account of the contribution of the conformational entropy of the core-forming block indicate that increasing branching of block *B* leads to a decrease in the micelle aggregation number and an increase in CMC, which is more pronounced in the crew-cut domain.

### 5.3. Comparison to Molecular Dynamics Simulations

Recent coarse-grained MD simulations [18] of AB diblock self-assembly in solution demonstrated how aggregation number *p* of micelles depends on the architectural parameters of comb-shaped blocks. Diblock copolymers with symmetric composition *A*:*B* = 50:50 were studied at fixed total diblock DP N=96, equal and variable lengths of side chains in both blocks, $n_{i}=n$, and equal lengths of spacers, $m_{i}=1$, in the backbone with DP M=N/(1+n). A set of 9 different block copolymer architectures was constructed via changing *n*, ranging from bottlebrush to starlike macromolecules. The average parameters of micelles, formed with inferior solvent strength for *B*-block, were presented as a function of branching parameter, $g=(\bar{R_{br}^{2}})$$/(\bar{R_{lin}^{2}})$, specified as the ratio of gyration radii, $\bar{R^{2}}$, of the branched (subscript br), and linear (subscript lin) individual chains with the same DP in a good solvent.

To relate *g* to molecular parameters in our model, we use the asymptotic power law dependencies for the average end-to-end distance (expressed in units of monomer length) for bottlebrush and starlike polymers with spacer length m≃1 under good solvent conditions. In the mean filed approximation, the average size of macromolecule, $R_{br}$, is given by

(38) $$R_{br}≃\left\{\begin{array}{cc} n^{2/5}M^{3/5} & bottlebrush\;polymer\\ n^{3/5}M^{1/5} & starlike\;polymer \end{array}\right.$$

The first line in Equation (38) is obtained by balancing the elastic free energy of the stretched bottlebrush backbone (∼$R^{2}/M$) with the free energy of repulsive binary interactions (∼${\left(Mn\right)}^{2}/R^{3}$). For a starlike polymer, the elastic free energy accounts for stretching of *M* side chains (∼$MR^{2}/n$) while the contribution of binary interactions remains the same as for bottlebrush polymer (∼${\left(Mn\right)}^{2}/R^{3}$). The crossover between two asymptotes in Equation (38) occurs at $M=M_{*}≃n^{1/2}$.

For a linear chain with *N* monomer units,

(39) $$R_{lin}≃N^{3/5}=M^{3/5}{\left(1+n\right)}^{3/5}\approx M^{3/5}n^{3/5}$$

The theoretical branching ratio *g* is then specified as

(40) $$g={\left(\frac{R_{br}}{R_{lin}}\right)}^{2}≃\left\{\begin{array}{cc} n^{-2/5} & bottlebrush\;polymer\\ M^{-4/5}={\left(n/N\right)}^{4/5} & starlike\;polymer \end{array}\right.$$

with the accuracy of prefactors on the order of unity. As it follows from Equation (40), an increase in *n* at fixed *N* leads to a non-monotonic dependence for g(n), in agreement with the simulation data (Figure 2g in Ref. [18]). The crossover between asymptotes for bottlebrush and starlike polymers occurs at

$$n_{*}≃N^{2/3};\;g_{*}=\;g\left(n_{*}\right)≃N^{-4/15}$$

For N=96, and both prefactors equal to unity in Equation (40), $n_{*}\approx 21$ and $g_{*}\approx 0.30$.

In selective solvent, AB copolymer with equal DPs of linear blocks ($n_{i}=0$) gives rise to starlike micelles in which the core of insoluble *B*-blocks is decorated by the corona of soluble *A*-blocks. As it follows from the diagram of states in Figure 3, branching of the blocks, i.e., an increase in $n_{A}$ at fixed value of $N_{A}$, leads to the non-monotonic variation in micelle aggregation number, *p*. According to Equation (32) in spherical micelles formed by diblock copolymer with $m_{A}/q_{A}≃1$, and $N_{B}=N_{A}=N/2$, the average aggregation number varies as

(41) $$p∼N_{B}^{10/11}\cdot \left\{\begin{array}{cc} N_{A}^{-3/11}{\left(1+n_{A}\right)}^{-9/11} & bb/st\\ N_{A}^{-21/11}n_{A}^{18/11} & mks/st \end{array}\right.=\left\{\begin{array}{cc} {\left(N/2\right)}^{7/11}{\left(1+n_{A}\right)}^{-9/11} & bb/st\\ {\left(N/2\right)}^{-1}n_{A}^{18/11} & mks/st \end{array}\right.$$

That is, aggregation number decreases from $p\left(n_{A}=0\right)=p_{lin}∼{\left(N/2\right)}^{7/11}$ in bb/st regime, reaches a minimum at the boundary between bb/st and mks/st regimes ($n_{A}≃N^{2/3}$), and subsequently increases back to $p_{lin}$ at $n_{A}=N/2$ in mks/st regime. By substituting g(n) from Equation (40) in Equation (41), one finds

(42) $$p≃p_{lin}\left\{\begin{array}{cc} g^{45/22} & bb/st\\ g^{45/22} & mks/st \end{array}\right.$$

Therefore, according to Equation (42), aggregation number *p* in micelles formed by copolymer with fixed DP *N* and bottlebrush blocks is expected to decrease upon an increase in *n* (due to decreasing *g*, Equation (40)), while for block copolymer with starlike blocks, *p* is expected to increase with *n* (and g), making the dependence p(g) looplike. The loop originates at g=1, and turns backwards at $g=g_{*}$. In the mean field approximation, the exponent in Equation (42) is the same for bottlebrush and starlike polymers, however, the omitted numerical coefficients and nonpower law dependencies could be different.

The predicted trends are in agreement with the results of MD simulations [18]. The latter demonstrated clear difference in the behavior of AB copolymers with bottlebrush (samples 1–6) and starlike (samples 7–9) blocks. In accordance with Equations (40) and (42), the data for micelle aggregation number $<N_{agg}>$ formed a loop as a function of branching parameter *g* (Figure 3e in Ref. [18]). The crossover value of $n_{*}≃21$ (estimated with accuracy of prefactors on the order of unity) was between DPs $N_{sc}=15$ and $N_{sc}=23$ in starlike samples 8 and 9 (Figure 3a in Ref. [18]). The crossover value of $g_{{}_{*}}≃0.30$ was close to g≈0.3, corresponding to reverse in $<N_{agg}>\left(g\right)$ dependence in MD simulations. However, evaluation of exponents in the asymptotic theoretical dependencies requires more extensive simulations with longer chains, and remains a challenge at the moment.

## 6. Conclusions

To summarize, we have developed a theory of micellization of diblock copolymers comprising chemically different comb-shaped (bottlebrush) blocks in selective solvents. This theory enables predicting how DPs of the main and side chains and grafting densities in both soluble and insoluble blocks of the copolymers affect aggregation number and the equilibrium dimensions of self-assembled micelles and critical micellar concentration. Both limits of blocks with long main chain and multiple short side chains (bottlebrush) and short main chain and a few long side chains (miktostar) are considered. Asymptotic analytical dependencies are derived for the limiting cases of starlike and crew-cut micelles, whereas full solution is obtained for arbitrary ratios between the micellar core and corona dimensions.

We have demonstrated that the replacement of the linear soluble block by a bottlebrush one with the same degree of polymerization results in a decrease in the aggregation number and dimensions (core size, corona thickness, the overall radius) of micelles. A similar and even stronger effect on *p* and *R* is predicted when the DP $M_{A}$ of the main chain of the soluble block is kept constant and the number and the DP $n_{A}$ of the side chains is increasing; in the latter case *D* is an increasing function of $n_{A}$.

As long as the main chain of the *A*-block is sufficiently long (the *A*-block has the bottlebrush shape), all structural properties of the micelles and CMC follow approximately power law dependencies on effective grafting density $q_{A}n_{A}/m_{A}$. When the DP $M_{A}$ of the main chain of the soluble block is short enough (miktostar regime), all these properties become a power law function of the number $M_{A}q_{A}/m_{A}$ of side chains in *A*-block.

The described effects of architecture of the soluble blocks on the properties of micelles are in full agreement with the trends observed in Molecular Dynamics simulations in Refs. [17,18] and can be explained by enhanced repulsive interactions and larger conformational entropy penalty for stretching of the comb-shaped blocks in the micellar corona, as compared to those for linear blocks. Experimental validation of these theoretical predictions would require comprehensive study of micellization of block copolymers with systematically varied DPs of the main and side chains in the soluble blocks

Replacement of the insoluble linear block by comb-shaped ones with the same DP $N_{B}$ results in a weak decrease in the aggregation number with concomitant decrease in the micellar size and increase in CMC. However, these dependencies are not described by power law functions. These effects are due to larger conformational entropy penalty for radial stretching of branched insoluble block in the micellar core. On the contrary, increasing DP and grafting density of side chains in the insoluble block with constant length of the main chain $M_{B}$ leads to an increase in $N_{B}$ and, as a consequence, increasing aggregation number and decreasing CMC.

Importantly, in the present study, we used a mean-field approximation, which is justified for semi-dilute solutions of semi-flexible polymers, that is, under the conditions that Kuhn segments of the main and side chains of both blocks are larger than the monomer unit size [24,25]. A more refined scaling analysis [7] may lead to different values of power law exponents for different properties of the block copolymer micelles. Such analysis, however, is beyond the scope of the present paper.

Finally, we remark that in the present study we considered only spherical micelles. By adjusting the sets of architectural parameters of the blocks, nano-aggregates of other morphologies (i.e., wormlike micelles, polymersomes) can be obtained as thermodynamically equilibrium structures. We shall address polymorphism of nanostructures formed by diblock copolymers with bottlebrush blocks in our forthcoming publication. 

## Data Availability

Not applicable.
